# Molecular Design of a Metal-Nitrosyl Ferroelectric with Reversible Photoisomerization

**DOI:** 10.1021/jacs.3c01530

**Published:** 2023-06-17

**Authors:** Wei-Jian Xu, Mao-Fan Li, Ana R. Garcia, Konstantin Romanyuk, José M. G. Martinho, Pavel Zelenovskii, Alexander Tselev, Luís Verissimo, Wei-Xiong Zhang, Xiao-Ming Chen, Andrei Kholkin, João Rocha

**Affiliations:** † Department of Chemistry & CICECO-Aveiro Institute of Materials, University of Aveiro, 3810-193 Aveiro, Portugal; ‡ MOE Key Laboratory of Bioinorganic and Synthetic Chemistry, School of Chemistry, Sun Yat-Sen University, Guangzhou 510275, China; § Department of Physics & CICECO-Aveiro Institute of Materials, University of Aveiro, 3810-193 Aveiro, Portugal; ∥ Centro de Química Estrutural, Institute of Molecular Sciences and Department of Chemical Engineering, Instituto Superior Técnico, University of Lisbon, 1049-001 Lisbon, Portugal

## Abstract

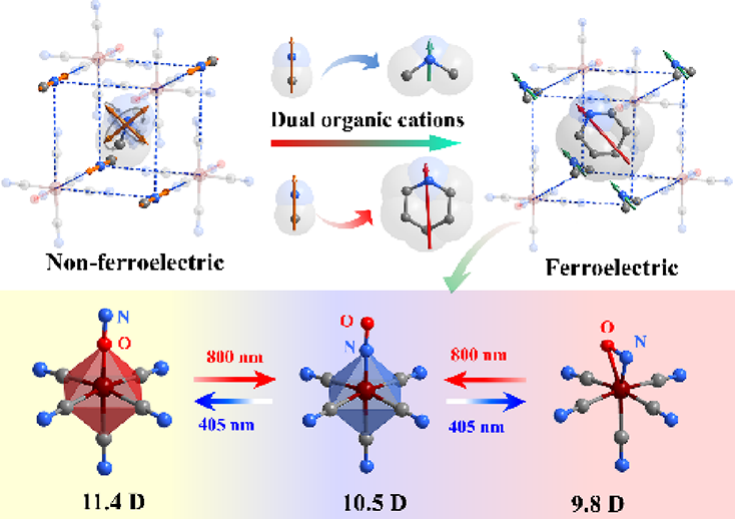

The development of photo-responsive ferroelectrics whose polarization may be remotely controlled by optical means is of fundamental importance for basic research and technological applications. Herein, we report the design and synthesis of a new metal-nitrosyl ferroelectric crystal (DMA)(PIP)[Fe(CN)_5_(NO)] (**1**) (DMA = dimethylammonium, PIP = piperidinium) with potential phototunable polarization via a dual-organic-cation molecular design strategy. Compared to the parent non-ferroelectric (MA)_2_[Fe(CN)_5_(NO)] (MA = methylammonium) material with a phase transition at 207 K, the introduction of larger dual organic cations both lowers the crystal symmetry affording robust ferroelectricity and increases the energy barrier of molecular motions, endowing **1** with a large polarization of up to 7.6 μC cm^–2^ and a high Curie temperature (*T*_c_) of 316 K. Infrared spectroscopy shows that the reversible photoisomerization of the nitrosyl ligand is accomplished by light irradiation. Specifically, the ground state with the N-bound nitrosyl ligand conformation can be reversibly switched to both the metastable state I (MSI) with isonitrosyl conformation and the metastable state II (MSII) with side-on nitrosyl conformation. Quantum chemistry calculations suggest that the photoisomerization significantly changes the dipole moment of the [Fe(CN)_5_(NO)]^2–^ anion, thus leading to three ferroelectric states with different values of macroscopic polarization. Such optical accessibility and controllability of different ferroelectric states via photoinduced nitrosyl linkage isomerization open up a new and attractive route to optically controllable macroscopic polarization.

## Introduction

The ability to control polarization states of ferroelectric materials with optical stimulation holds much promise for modern technological applications, such as optically driven mechanical actuators, optical information storage, and optically-addressed ferroelectric memories.^1−3^ Consequently, over the past decades, considerable efforts have been made toward the optical manipulation of ferroelectric properties at the microscopic and macroscopic scales.^4^ For instance, the photomodulation of spontaneous electric polarization has been achieved in ferroelectric liquid crystals via photochemical processes, such as *cis*–*trans*, and open/closed-ring isomerization.^5−7^ Remarkably, light-induced local polarization switching and domain-wall motion have been observed in inorganic ferroelectrics through the photoexcited thermal (e.g., thermoelectricity and pyroelectricity) and electronic (e.g., photovoltaic and photoinduced flexoelectricity) effects,^8−11^ not to mention photoinduced poling and domain manipulation in ferroelectric thin films.^12,13^

By virtue of their high structural tunability and versatility, solid-state molecule-based ferroelectrics and pyroelectrics are promising platforms for the optical manipulation of ferroic orders.^14−17^ Significant breakthroughs in this field have been made by the discovery of a correlation between the ferroelectric properties and light–triggered structural changes in a series of photochromic pure organic ferroelectric crystals.^18−24^ For instance, Xiong et al. reported the photoinduced polarization switching in salicylideneaniline-based ferroelectric crystals through light-driven enol-keto geometrical isomerization witnessed by reversible ferroelectric domain changes under illumination.^22^ While the attention has been mostly focused on local effects under illumination,^10^ the macroscopic effects are much less studied, experimentally and theoretically.^4,25−29^ Recently, Sato et al. demonstrated the macroscopic polarization change in polar valence tautomeric/spin transition complexes via light-induced electron transfer/ion displacement.^30−32^ Nevertheless, research on photo-controllable polarization of molecular ferroelectrics/pyroelectrics materials is still in its infancy, with fundamental and applied issues deserving attention, including low spontaneous polarization,^22^ transient lifetime of metastable states,^33,34^ irreversible switching between different metastable states,^19^ and low photo-conversion efficiency.^35^ In this context, it is a prerequisite to explore new photo-responsive molecular ferroelectrics, particularly those exhibiting large spontaneous polarization.

A family of metal-nitrosyl (M–NO) complexes is a very promising photoresponsive system,^36,37^ comprising sodium nitroprusside, where the light-induced interconversion between the ground state (GS) and two long-lived (τ > 10^7^ s) metastable states, MSI and MSII, occurs at specific irradiation wavelengths or is prompted by temperature. Certain types of structural isomerization, such as the enol–keto isomerization in Schiff bases, *cis*–*trans* in azo, and open/closed ring in spiropyran and diarylethene derivatives, require a large free space in the solids. In contrast, the small nitrosyl ligand size allows easy photoexcitation to metastable isomers without a pronounced photomechanical effect.^38^ More importantly, computational and experimental investigations have shown that photoswitching between GS and MSI/MSII states induces a significant hyperpolarizability change.^39−41^ This provides an important clue to designing phototunable non-linear optical and M–NO ferroelectric complexes. Although the observation of the nitrosyl linkage photoisomerization in sodium nitroprusside was first reported in 1977,^42^ further research was mainly focused on the electronic and molecular structures and kinetic and thermodynamic properties of the metastable states,^43^ while the cross-coupling photophysical effects caused by such photoisomerization have been rarely studied.^44,45^

**Figure 1:**
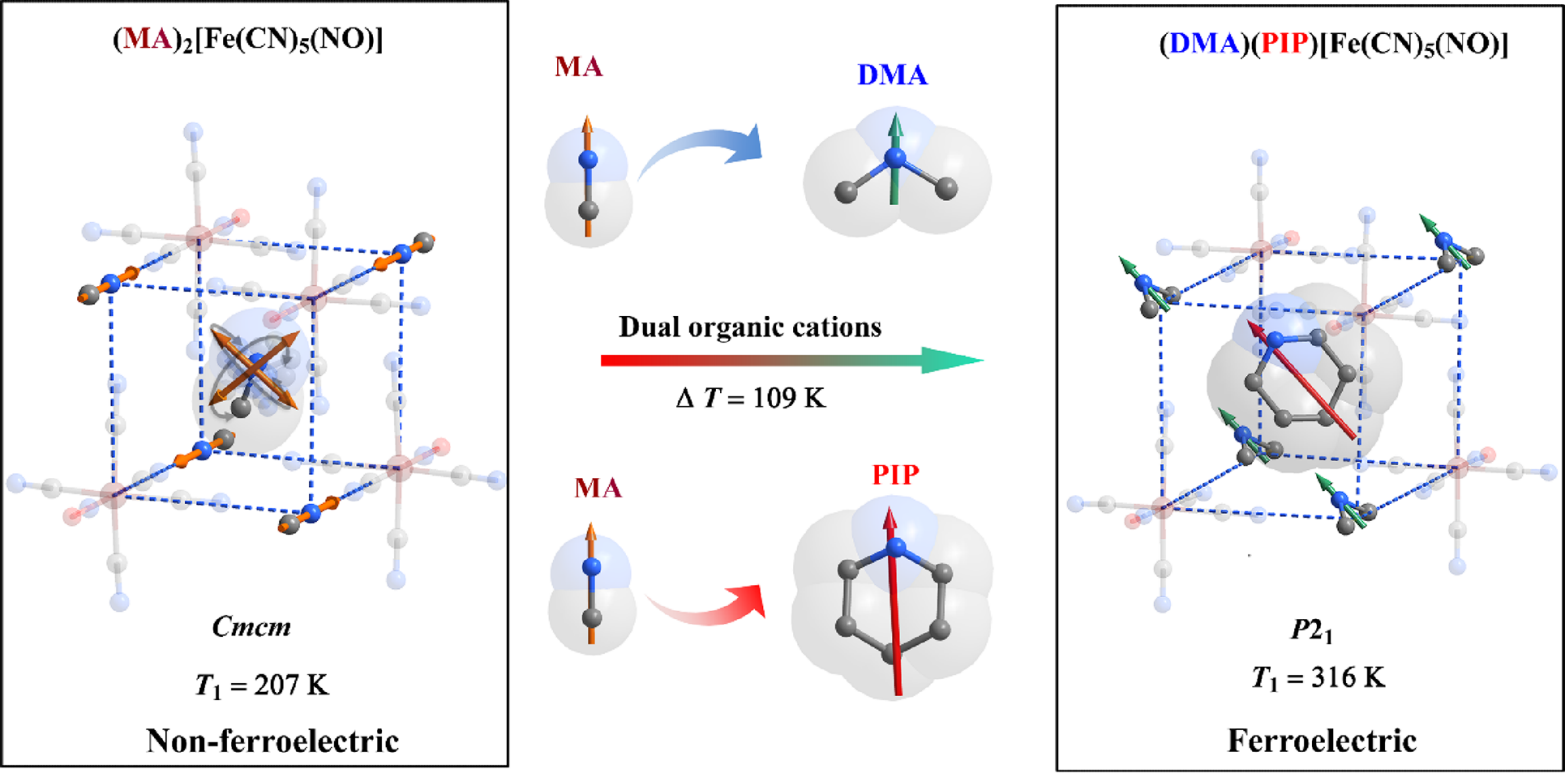
Design concept and strategy of nitroprusside-based ferroelectric (DMA)(PIP)[Fe(CN)_5_NO] **1**. MA, methylammonium; DMA, dimethylammonium; PIP, piperidinium. Arrows denote the permanent dipole moments of the organic molecules. Hydrogen atoms are not shown, for clarity.

Recently, several reports have demonstrated that the co-assembly of various organic cations with the nitroprusside anion [Fe(CN)_5_(NO)]^2–^ produces a new class of organic–inorganic hybrid materials with diverse structures and fascinating physical properties.^46−52^ For instance, Yao et al. reported a *Pbcm* to *Cmcm* phase transition at 207 K in (MA)_2_[Fe(CN)_5_(NO)] (MA = methylammonium).^49^ However, the antiparallel arrangement of polar MA cations and their dynamical disordering cancel out the total unit cell dipole moment, preventing the emergence of long-range order and electric polarization (Figure 1, left). Inspired by the “ferroelectrochemistry” and organic-cation engineering,^53−58^ we have broken the symmetry of the (MA)_2_[Fe(CN)_5_(NO)] unit cell by replacing the MA cations with dimethylammonium (DMA) at the corners, and a larger-size piperidinium (PIP) cation in the cage (Figure 1, right). In the ensuing new complex, (DMA)(PIP)[Fe(CN)_5_(NO)] (**1**, Scheme S1), the orientational order of the organic cations stabilized by steric effects and strong hydrogen bonds results in room-temperature ferroelectric properties. In particular, the presence of large organic cations increases the energy barriers for the molecular motion in **1**, leading to a Curie temperature of 316 K, i.e., 109 K higher than the parent compound (MA)_2_[Fe(CN)_5_(NO)]. Moreover, due to the dual contribution of the dipole moments of the organic cations, **1** exhibits a saturated polarization, up to 7.6 μC cm^–2^, that is four times larger than the first reported nitroprusside-based ferroelectric (DMA)[NaFe(CN)_5_(NO)] bearing a mono organic cation.^48^ Such a dual-organic-cation strategy shows that controlling the molecular orientation to tune the symmetry of nitroprusside-based crystals and optimizing their polarization enable the design of new high-performance molecular ferroelectrics.

Intriguingly, upon irradiation with alternating blue and red lights, **1** exhibits the reversible photoswitching of the nitrosyl linkage isomers between GS, with the N-bound nitrosyl conformation, and two long-lived metastable states: MSI, with isonitrosyl conformation, and MSII, with side-on nitrosyl conformation, as revealed by IR spectroscopy. According to quantum-chemical calculations, such unique photoisomerization induces a large change in the dipole moment of nitroprusside anions, leading to three ferroelectric states with different macroscopic polarization values.

## Results and Discussion

### Preparation and Phase Transitions

**Figure 2:**
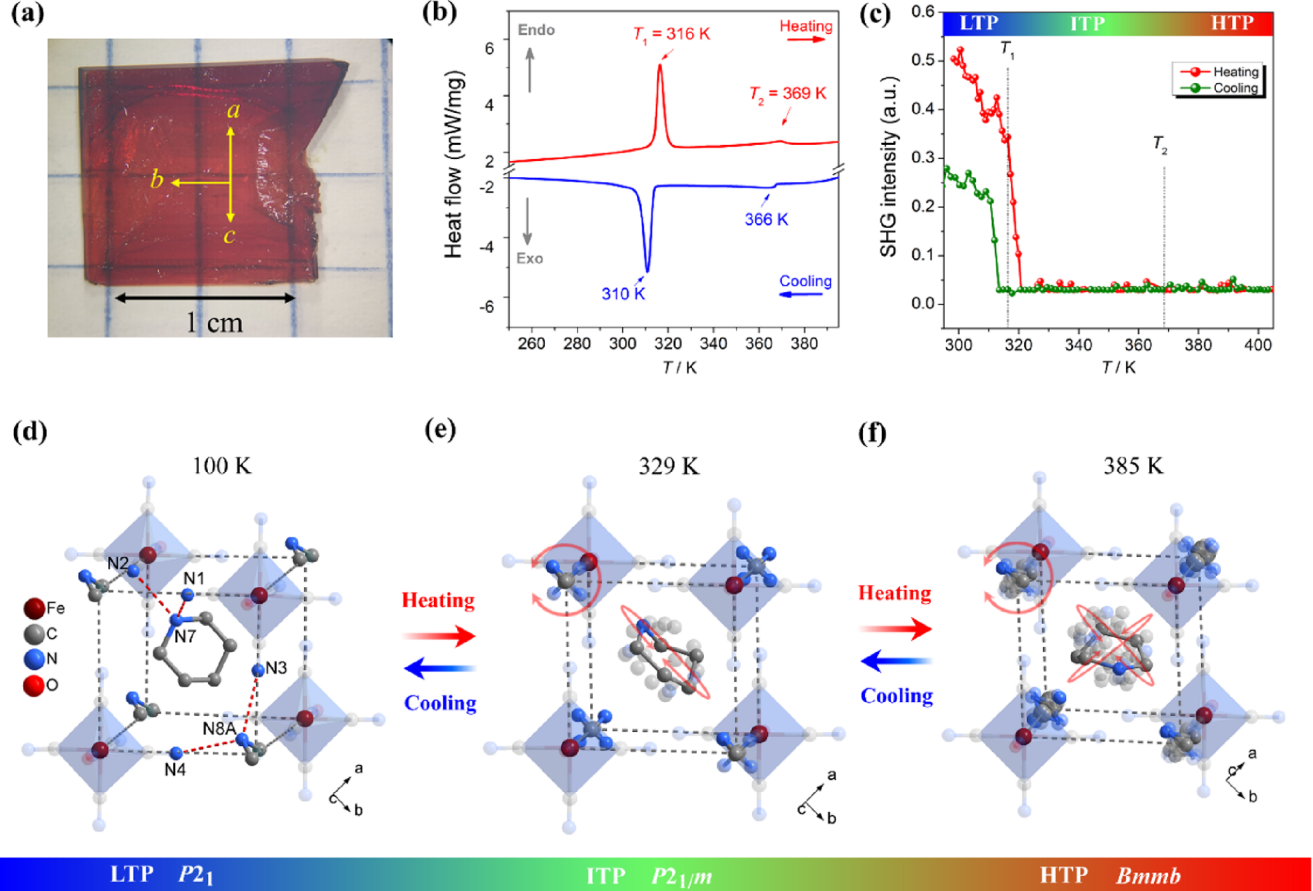
(a) Photograph of a single crystal of **1**. (b) Differential scanning calorimetry curves of **1** measured on a powdered sample in heating and cooling runs. (c) Second harmonic generation intensity as a function of temperature of **1** measured on a powdered sample. Crystal structures of **1** at (d) LTP (100 K), (e) ITP (329 K), and (f) HTP (385 K). Only one PIP and one DMA cation are highlighted for ITP and HTP. Hydrogen atoms are not shown, for clarity.

Centimeter-size high-quality single crystals of **1** were prepared by the slow evaporation method (Figure 2a, Supporting Information). Powder X-ray diffraction and elemental analysis confirmed the phase purity of the polycrystalline powder (Figure S2). Thermogravimetric analysis revealed that **1** is stable up to ∼440 K (Figure S3). Differential scanning calorimetry showed two pairs of reversible anomalies at 316/310 K (*T*_1_) and 369/366 K (*T*_2_) in heating/cooling runs, suggesting that **1** undergoes two reversible structural phase transitions (Figure 2b). The corresponding entropy change Δ*S* for the heating mode was estimated to be 18.8 and 1.26 J mol^–1^ K^–1^ at *T*_1_ and *T*_2_, respectively. For convenience, we label the phase below *T*_1_ as the low-temperature phase (LTP), between *T*_1_ and *T*_2_ as the intermediate-temperature phase (ITP), and above *T*_2_ as the high-temperature phase (HTP).

The symmetry breaking during the phase transitions was identified by temperature-dependent second harmonic generation (SHG) measurements. As shown in Figure 2c, a noticeable SHG signal was observed below *T*_1_, suggesting for LTP a non-centrosymmetric structure. Above *T*_1_, the SHG intensity decreased to the noise level and remained unchanged up to 405 K. These observations indicate centrosymmetric structures for ITP and HTP.

### Crystal Structures

At 100 K (LTP), **1** crystallizes in the monoclinic space group *P*2_1_, with cell parameters *a* = 9.0562(8) Å, *b* = 9.0881(9) Å, *c* = 10.7238(9) Å, β = 107.767(3)°, and *V* = 840.51(13) Å^3^ (Table S2). The asymmetric unit consists of one [Fe(CN)_5_(NO)]^2–^ anion, one DMA cation, and one PIP cation. **1** may be described as a two-dimensional supramolecular cage-like structure (Figure S4), bearing similarity with (MA)_2_[Fe(CN)_5_(NO)].^49^ Each cage comprises four [Fe(CN)_5_(NO)]^2–^ anionic octahedra and four DMA cations, with PIP cations residing in the central cavity (Figure 2d). Each PIP cation interacts with [Fe(CN)_5_(NO)]^2–^ via two hydrogen bonds (N7–H···N1 distance of 2.877(8) Å and N7–H···N2 distance of 2.918(8) Å), while the DMA cation forms two hydrogen bonds with [Fe(CN)_5_(NO)]^2–^ (N8A–H···N3 distance of 2.849(8) Å and N8A–H···N4 distance of 2.823(7) Å). These bond distances are much shorter than N–H···N (3.067 Å) in (MA)_2_[Fe(CN)_5_(NO)], pulling both organic cations toward the [010] direction. As a result, the ordered alignment of the organic cations creates a spontaneous polarization along the *b*-axis. At 298 K, DMA cations display orientational disorder over three sites with N-atom occupancies 0.62, 0.25, and 0.13. (Table S2).

At 329 K (ITP), **1** still crystallizes in the monoclinic system but in the non-polar space group *P*2_1_/*m* (Table S2). The DMA cations dynamically rotate and are disordered over four sites (Figure 2e). Likewise, PIP cations show a fourfold disorder, the combination of rotational and oscillating motions of the ring (Figure 2e). According to the symmetry breaking analysis, LTP **1** belongs to ferroelectric species with Aizu notation 2/*m*F2,^59^ i.e., there are four symmetric elements in ITP (*E*, *C*_2_, *m*, *i*), which are reduced to two (*E*, *C*_2_) in the ferroelectric LTP.^59^ The paraelectric-ferroelectric transition from ITP to LTP is of the order–disorder type. A similar paraelectric-to-ferroelectric phase transition of 2/*m*F2 type was observed in other molecular-based ferroelectrics, such as (DMA)[Mn(N_3_)_3_],^60^ imidazolium periodate (IPI),^61^ 4-(cyanomethyl)anilinium perchlorate,^62^ and diisopropylammonium bromide (DIPAB).^63^

Above *T*_2_ (HTP), **1** transforms into the orthorhombic centrosymmetric space group *Bmmb* (an alternative notation for the space group *Cmcm* (No. 63) that was chosen to facilitate the comparison of the different phase structures). As shown in Figure 2f, the DMA rotational motion and the PIP in-plane and out-of-plane motions lead to eightfold crystallographic disorder with respect to two mirror planes perpendicular to the *a* and *b* axes, respectively. The HTP cell volume is almost twice the ITP one. The HTP-to-ITP transition is ferroelastic with Aizu notation *mmm*F2/*m*, which generates two ferroelastic species (twins) in the ITP phase with the permissible ferroelastic (twin) domain walls along the monoclinic twofold axis of the ITP phase. In turn, the IPT-to-LTP transition is ferroelectric, but not ferroelastic, where the monoclinic twofold axis (*b*-axis) becomes polar and no more ferroelastic species are produced. Hence, the orthorhombic phase is structurally the prototype phase for both ITP and LTP with the two-step transition to the ferroelectric phase. In short, such two-step structural phase transitions are ascribed, mainly, to the orientational order–disorder transition of DMA and PIP cations, whereas the conformation of the nitroprusside anions remains almost unchanged.

### Dielectric and Ferroelectric Properties

**Figure 3:**
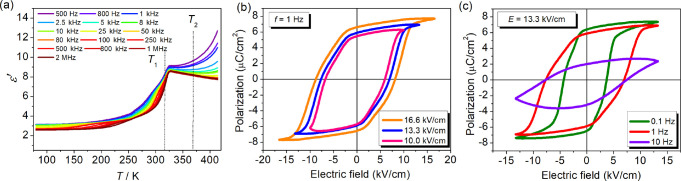
(a) Temperature-dependent real part (ε′) of the complex dielectric constant measured on the polycrystalline pellet of **1** at various frequencies during heating. Electric-field-dependent (b) and frequency-dependent (c) ferroelectric hysteresis loops measured on a single crystal of **1** parallel to the *b*-axis, at 298 K.

The temperature-dependent complex permittivity (ε = ε′ – *i*ε″, where ε′ and ε″ are the real part and imaginary parts, respectively) measurements were performed on a pressed powder pellet of **1**. Upon heating, the real part (ε′) of the complex dielectric constant exhibited a (slight) frequency-dependent increase, followed by a sharp jump in the vicinity of *T*_1_ = 316 K (Figure 3a). No dielectric anomaly was observed at *T*_2_, due to only a slight change of dipole moment reorientation between ITP and HTP, witnessed by the aforementioned structural analysis. Notably, ε″ shows two distinct dielectric relaxation processes in the frequency range below *T*_1_ (Figure S5), ascribed to the dynamic rotational motion of the DMA cation in LTP. The dielectric response is reproducible under a reverse cooling process (Figure S6), confirming the intrinsic orientational polarization of the polar organic molecules.^64^

To demonstrate the room-temperature bulk ferroelectricity of **1**, electric polarization versus electric field (*P*–*E*) hysteresis loops were measured on a single crystal using a Sawyer–Tower circuit, with varying electric fields parallel to the *b*-axis (Figure S7). As shown in Figure 3b, a well-defined rectangular loop with a maximum saturated polarization (*P*_s_) of 7.6 μC cm^–2^ and a coercive field (*E*_c_) of 8.1 kV/cm were observed for applied AC electric fields up to 16.6 kV/cm at frequency 1 Hz. The *P*–*E* loops measured at an electric field of 13.3 kV/cm and frequencies in the range of 0.1–10 Hz are shown in Figure 3c. At higher frequencies, the remnant polarization (*P*_r_) slightly decreases from 6.4 to 5.8 μC cm^–2^ whereas the coercive field doubles from 3.6 kV/cm at 0.1 Hz to 7.0 kV/cm at 10 Hz. The deformation of the *P–E* loop observed at 10 Hz is due to slow polarization reversal in **1** with increasing frequency.^65^

While the observed *E*_c_ values are typical of other molecular ferroelectrics,^66−70^ they are much lower than those of poly(vinylidene) (PVDF) (∼500 kV/cm)^71^ and (C_7_H_16_N_2_)(NH_4_)(PF_6_)_3_ (110 kV/cm).^28^ Another notable feature is that the *P*_s_ value is much larger than those of photoswitchable and dual-organic-cation-based ferroelectrics, such as a diarylethene derivative (1.3 μC cm^–2^),^23^*N*-salicylidene-2,3,4,5,6-pentafluoroaniline (0.84 μC cm^–2^),^18^ (CH_3_(CH_2_)_2_NH_3_)(MA)[SbBr_5_] (2.9 μC cm^–2^),^54^ and (DMA)(C_6_H_5_CH_2_NH_3_)_2_[BiBr_6_] (1.0 μC cm^–2^).^55^ Moreover, the *P*_s_ value of **1** is four times larger than that of the first discovered nitroprusside-based ferroelectric (DMA)[NaFe(CN)_5_(NO)] (1.65 μC cm^–2^),^48^ which is attributed to the additional contribution from the PIP dipole moment that is somewhat larger than DMA.

### Ferroelectric Domains and Polarization Reversal

**Figure 4:**
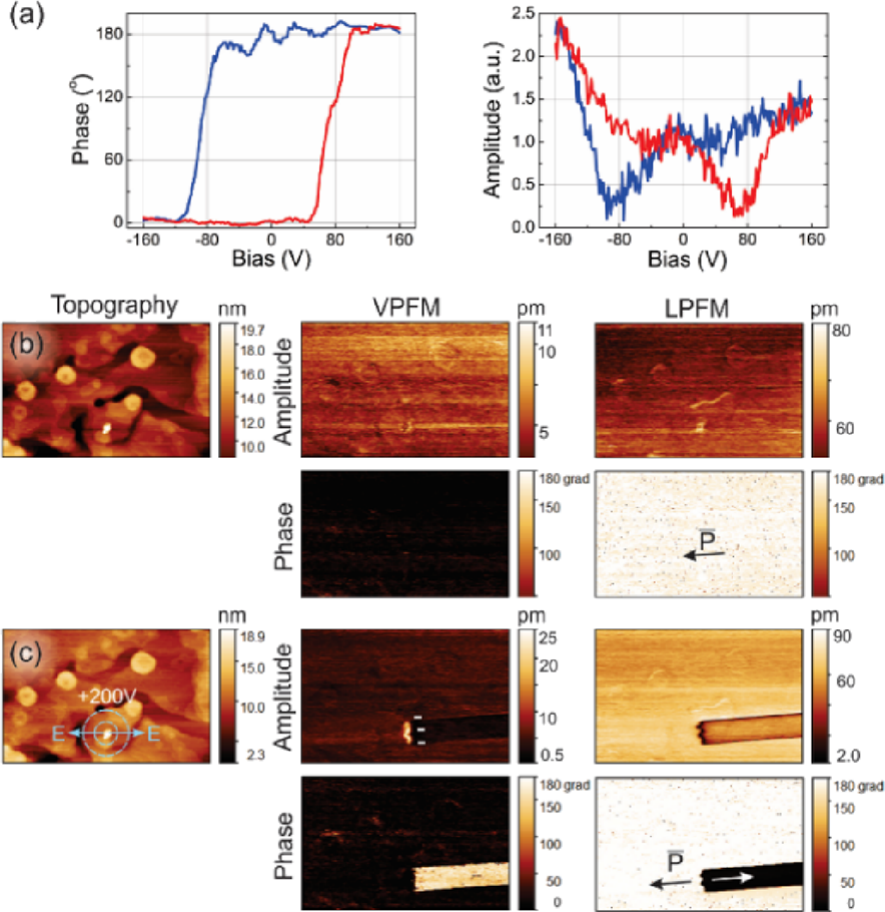
Domain switching measurements of an as-grown sample of **1**. (a) Switching spectroscopy of the phase (left) and amplitude (right). (b) Initial PFM images. (c) Local domain switching by application of a voltage +200 V for 10 s at the point marked by the arrows in the topography image. The polarization direction is depicted by arrows in the LPFM phase image. Image size is 20 × 12 μm^2^.

Piezoresponse force microscopy (PFM) was used to estimate the local piezoelectric response and to image apparent ferroelectric domains at the nanoscale for **1**.^72−75^ The PFM measurements were performed at room temperature on as-grown and annealed samples of **1**. The measured crystal plane (001) was verified by X-ray diffraction and the Bravais–Friedel–Donnay–Harker method (Figure S8). The switching spectroscopy PFM measurements on an as-grown crystal show the phase–voltage hysteresis loop and amplitude–voltage butterfly loop that indicates nanoscale switchable polarization (Figure 4a).

To visualize the domain switching behavior, DC bias was applied to the PFM probe, while the back electrode (underneath the sample) was grounded. Figure 4b shows as an initial state a scanned region of an as-grown sample with a single ferroelectric domain (at least, in the scanned area), which revealed a significantly stronger lateral PFM (LPFM) piezoelectric signal than the vertical one (VPFM) because of the apparent in-plane polarization. The application of a +200 V DC bias voltage to the probe generated an electric field, which had opposite signs in opposite directions from the probe-sample contact point on the sample surface. The voltage was applied for 10 s with the probe in contact with the sample at the selected point (see Figure 4c, topography). As seen in Figure 4c, the polarization was successfully switched in the part of the scanned area, which is indicated by the 180° PFM phase contrast between the two opposite polarization states in the phase images and the domain walls revealed in the amplitude images. It is worth noting that a domain wall with a negative polarization-bound charge (“tail-to-tail” wall) was created at the tip of the stripe-like domain, where polarization switched its direction. Bound polarization charges in the “head-to-head” and “tail-to-tail” domain walls induce an electric field, which is energetically costly and, therefore, should be screened by charges of the opposite sign. Such screening is evidenced by the lack of the signal in measurements in the Kelvin probe force microscopy (KPFM) mode performed with the probe out of contact with the sample over the tail-to-tail domain wall in Figure 4c. However, in the contact VPFM regime (Figure 4c, amplitude), a high electromechanical response was observed at the “tail-to-tail” domain wall. The phase of such response is in agreement with the electrostatic contribution expected from the negative charge of the “tail-to-tail” wall. The electric charge of the domain wall is opposite in polarity to the polarity of the probe; therefore, the effect of the charge injected into the sample from the probe can be ruled out. Screening of the domain wall charge can be due to mobile organic anions and cations present on the sample surface. We speculate that the surface conductivity can be associated with the presence of a wetting layer and partial dissolution of the crystal resulting in mobile organic anions and cations.

**Figure 5:**
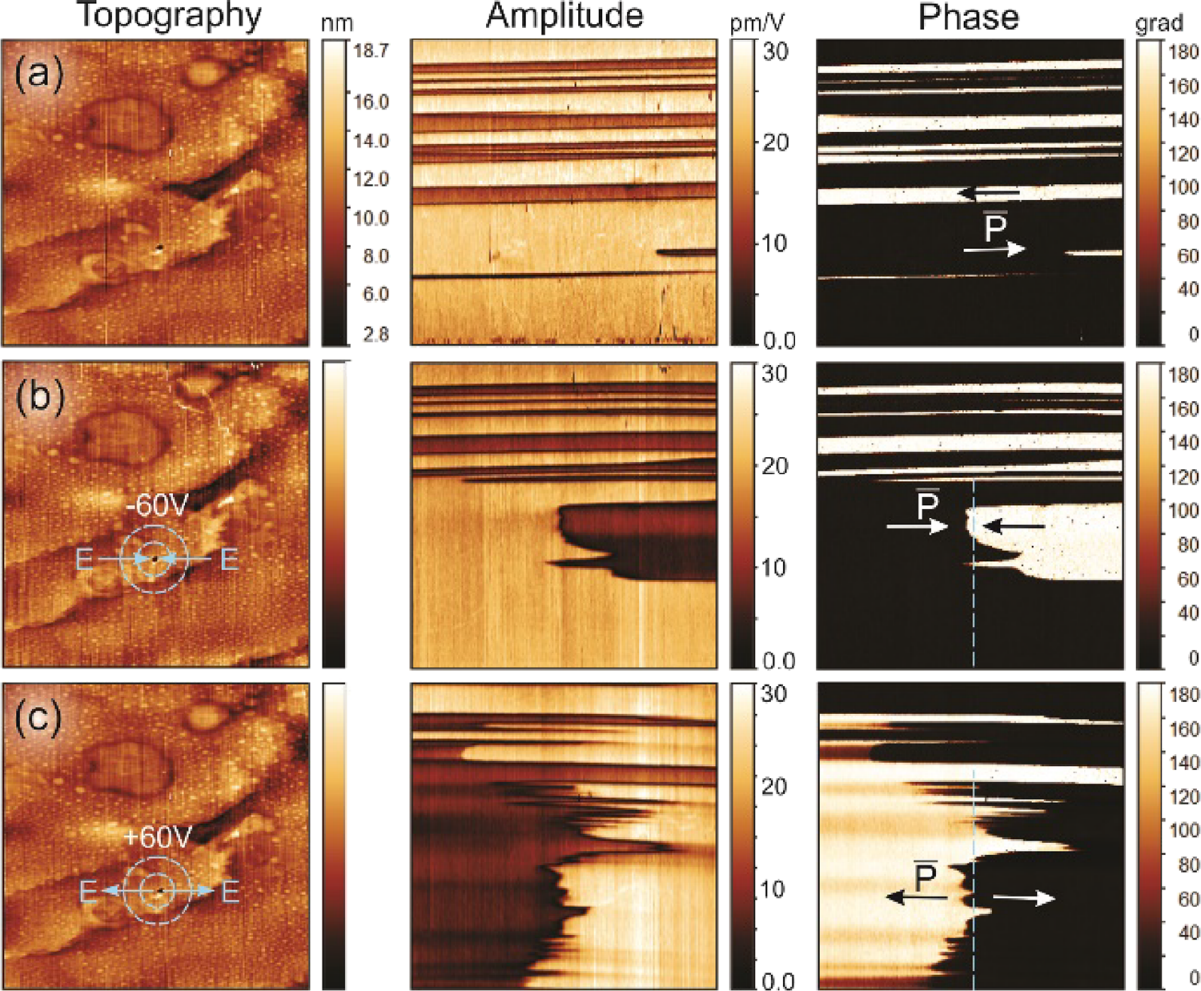
Domain switching measurements on a sample of **1** annealed at 343 K for 5 min. LPFM images of topography (left), amplitude (middle), and phase (right) images, which were observed in the initial state (a) and after applying a voltage of (b) −60 V and (c) subsequently +60 V at the selected position marked by the dashed circle in the topography images. Image size is 60 × 60 μm^2^.

Following the PFM measurements, the as-prepared sample was annealed at 343 K for 5 min. This annealing induced the ferroelectric–paraelectric phase transition from LTP to ITP and back. After this step, instead of a single domain, PFM revealed a system of alternating stripe-like ferroelectric domains with nearly parallel domain walls (Figure 5a, amplitude and phase images). The domain walls are parallel to the (polar) *b*-axis of the crystal and neutral, separating domains with opposite polarization directions. This observation is in accordance with the two-step transition *mmm*F2/*m* → 2/*m*F2 established in the analysis of the lattice structure of **1** and confirms the uniaxial ferroelectricity of **1**. The monoclinic ITP is ferroelastic (and paraelectric) and is naturally split into ferroelastic domains (twins) to accommodate mechanical stresses in the sample^59^ arising from the thermal annealing cycle. The neutral ferroelectric domain walls in the LTP (separating opposite polarizations) run along the polar axis, which is parallel to the walls of the ITP ferroelastic domains. In such a case, the neutral ferroelectric domain walls are mobile due to the lack of a significant energy barrier for their motion but can be pinned by ferroelastic domain walls. Hence, the domain geometry seen in the PFM images in Figure 5a reflects the ferroelastic domain structure in the imaged area. The twinning (separation into ferroelastic domains) is clearly seen in topography images of the samples annealed above *T*_2_, i.e., the temperature of the ITP-to-HTP transition (Figure S9).

To test the ferroelectric switching behavior of the annealed (and twinned) sample, a bias voltage of −60 V was applied to the PFM probe at a selected point for 10 s. After subsequent PFM imaging, a bias of +60 V was applied for 10 s at the same point. Following the voltage applications, the irregular changes in the domain pattern were observed with polarization switched to the opposite direction in a significant portion of the material within the imaged area (Figure 5b,c). The orientation of domain polarization obtained after voltage application is defined by the lateral components of the electric field at the probe-sample contact point. The dashed blue line in the LPFM phase images in Figure 5b,c separates two regions with opposite polarizations following electric field poling. A feature of these images is the highly rough, ragged ferroelectric domain walls between regions with switched polarizations that run on average along the dashed blue lines. These domain walls are “head-to-head” and “tail-to-tail” and, therefore, carry a non-zero-bound charge, which was also indicated by the high electromechanical response in the VPFM amplitude images (Figure S10). Such charged domain walls are energetically unfavorable as mentioned above, and the rough, ragged shape of the walls can be a mechanism invoked by the system to reduce the excess electrostatic energy of the walls. However, it can be stated that the material possesses intrinsic mechanisms for wall charge compensation (screening), e.g., mobile ionic charges, that stabilize the charged domain walls.^76,77^ The nature of these mechanisms, which may be specific to this organic–inorganic hybrid ferroelectric, requires additional studies beyond the scope of the current report.

### Photoisomerization

**Figure 6:**
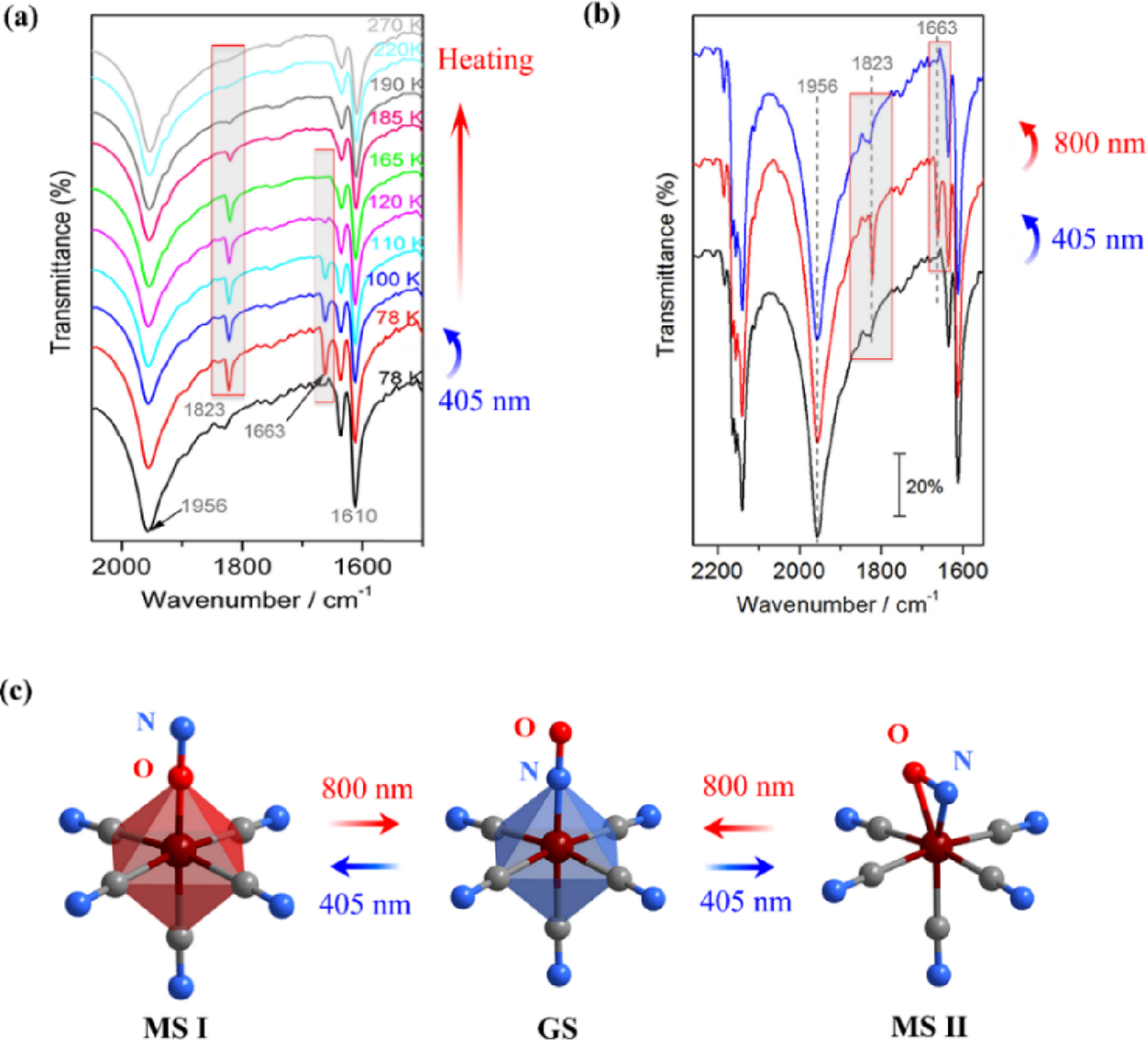
(a) Variable-temperature IR spectra of **1**. New absorption bands appear at 1823 and 1663 cm^–1^ following 405 nm light irradiation for 1 h at 78 K. (b) Photo-reversible change witnessed by the IR spectra upon 405 and 800 nm light irradiation at 78 K. (c) Reversible photo-induced structural isomerization of the nitrosyl ligand prompted by alternating 405 and 800 nm irradiation.

To investigate the photo-induced linkage isomerism in **1**, infrared (IR) spectra were measured before and after light irradiation at different temperatures (Figure S11). Figure 6a shows the IR spectra in the relevant spectral region of 2050–1500 cm^–1^ before (black curve) and after the sample irradiation at 405 nm for 1 h (color curves). In GS (i.e., before irradiation), the absorption band of the NO stretching vibration of nitrosyl ligand, ν(NO), is observed at 1956 cm^–1^. Upon photo-excitation at 405 nm, two new bands appear at 1823 and 1663 cm^–1^, attributed to ν(NO) vibrations of the metastable phases MSI and MSII, respectively. The irradiation promotes one electron from the 3d orbitals (3d_*zx*_, 3d_*yz*_, 3d_*xy*_) of the Fe^2+^ central atom to the empty antibonding orbital π* of NO. Subsequently, this intermediate state undergoes relaxation through non-radiative transitions, ultimately reaching the GS, metastable state I (MSI), and metastable state II (MSII). The intensity of these bands progressively diminishes upon heating, vanishing at approximately 190 and 120 K, in accordance with the potential barriers to the GS of 0.7 eV for metastable state I (MSI) and 0.5 eV for metastable state II (MSII), respectively. This indicates a temperature-induced reverse transformation to the GS for both metastable states. The MSI and MSII decay temperatures are comparable to the well-studied compound Na_2_Fe(CN)_5_(NO)·H_2_O.^78^

To verify the reversibility of photo-induced isomerization of the nitrosyl ligand, the IR spectra were also recorded after alternating irradiation of the sample with blue (405 nm) and red (800 nm) lights. As shown in Figure 6b, the ν(NO) absorption bands of MSI and MSII states (1823 and 1663 cm^–1^, respectively) appear after blue light irradiation (405 nm) for 50 min and vanish following red light irradiation (800 nm) for 5 min, indicating a completely reversible transformation back to GS (Figure 6c). Upon irradiation with 800 nm light, the π* (NO) orbital of both MSI and MSII states is excited, resulting in the formation of an intermediate state. This intermediate state subsequently relaxes non-radiatively back into GS.

Based on the intensity of the GS ν(NO) bands before and after irradiation, the fraction of [Fe(CN)_5_(NO)]^2–^ anions converted from GS to MSI and MSII is estimated to be ca. 3%. This population depends much on external experimental factors, such as incident light intensity, sample preparation, irradiation time, and also inherent lattice strains during the isomerization process.^79^ The dual-organic-cation strategy presented in this work affords chemical flexibility to control the crystal packing using different sizes and symmetric organic cations, to reduce the unfavorable strain and improve the conversion percentage.^80^ Future work will aim to increase the yield of the metastable phases through the optimization of experimental parameters.

### Quantum-Chemical Calculations

**Figure 7:**
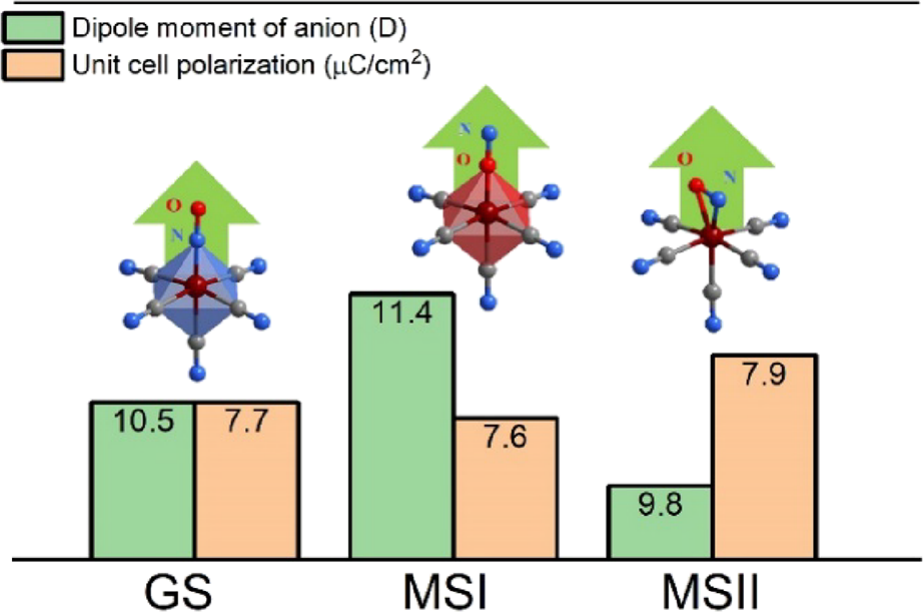
The calculated dipole moments of an individual nitroprusside anion on different metastable states and the total polarization of the unit cell. The calculations were performed for the LTP unit cell at 100 K. Green arrows show the direction of the dipole moment of the nitrosyl anions.

The transition from GS to the metastable phases involves the reorganization of the Fe–N–O bond in the [Fe(CN)_5_(NO)]^2–^ anion. In GS, this bond is linear, whereas N–O rotates by 180 and 85° in MSI and MSII, respectively.^81^ Therefore, the photoisomerization may, in principle, affect the polar properties of **1**. Quantum-chemical calculations estimated a polarization along the polar *b*-axis of 7.3 μC/cm^2^ for the GS unit cell at 298 K (Table S1), comparable to the experimental value (7.6 μC/cm^2^). Since the orientation of the DMA cation dipole moment is mainly associated with the nitrogen atom (Figure S1d), the ordering of DMA cations at lower temperatures results in a polarization increase up to 7.7 μC/cm^2^ at 100 K. Notably, the dipole moment of an individual nitroprusside anion significantly changes after the transition from GS to one of the metastable states. In MSI, the anion’s dipole moment is around 8% higher than in GS, whereas in MSII, it is about 6% lower (Figure 7). The orientation of the dipole moment does not change and remains aligned with the Fe–N–O bond (Figure 7). If both anions in the unit cell at GS are transferred to the MSII state, the polarization increases from 7.7 to 7.9 μC/cm^2^ (Figure 7). Likewise, the polarization value of the MSI state is estimated to be 7.6 μC/cm^2^. When the 3% [Fe(CN)_5_(NO)]^2–^ anion is photo-excited to both MSI and MSII, the total polarization of the unit cell of GS decreases to 7.4 μC/cm^2^.

The polar order arises from the collective orientation of dual organic cations, while the nitroprusside anions with opposite orientations lead to partial compensation of the total dipole moment. Therefore, it is expected that phototunable ferroelectric polarization with sufficiently high contrast may be potentially achieved through an appropriate alignment of the dipole moment of the nitroprusside anion that contributes to polarization. We note that the photoconversion efficiency needs to be improved for the experimental observation of polarization change.

Nevertheless, our experimental and computational results demonstrate that the three ferroelectric states with different polarization strengths are optically accessible and controllable via nitrosyl photoisomerization, thus providing an attractive possibility for modulating the strength of the macroscopic ferroelectric polarization.

## Conclusions

In summary, we have reported the design and synthesis of a new photo-responsive metal-nitrosyl ferroelectric **1** exhibiting a large polarization, up to 7.6 μC cm^–2^, and a high Curie temperature of 316 K. The dual-organic-cation strategy is a powerful design principle to realize electrical ordering and optimize the ferroelectric performance, prompting the development of molecular ferroelectrics. More generally, benefiting from the great variety of organic cations available, it is expected that a large family of metal-nitrosyl complexes with the general formula (A)(A′)[Fe(CN)_5_(NO)] (A and A′ = mono-valent organic cation) may be rationally synthesized through the combination of structural and chemical degrees of freedom. More importantly, our experimental and computational results showed that the phototunable polarization of **1** might be potentially accomplished via photoinduced nitrosyl linkage isomerization. Such optical accessibility and controllability of different ferroelectric states open up new intriguing routes for the optical manipulation of macroscopic polarization. We envision that these results will inspire further research on new photo-responsive molecular ferroelectrics with photo-induced linkage isomerization based on other small ambidentate ligands, such as CN^–^,^82^ SO_2_,^83^ SCN^–^,^84^ and NO_2_^–^.^85^

## Supporting Information Available

Experimental section including synthesis, measurement methods of X-ray diffraction, dielectric, *P*–*E* hysteresis loop measurements, thermal analysis, DSC, SHG, infrared spectroscopy, and PFM measurements; PXRD patterns; crystal structures; dielectric response; crystal morphology; TGA and DTA thermograms; table of crystal data and structure refinement parameters (PDF)
