# Supplementary material for: Molecular Design of a Metal-Nitrosyl Ferroelectric with Reversible Photoisomerization
Source: J Am Chem Soc. 2023 Jun 17;145(25):13663–73. doi: 10.1021/jacs.3c01530 (PMC10311532; doi:10.1021/jacs.3c01530)
Supplement: Supplementary file 1 — ja3c01530_si_001.pdf [file ja3c01530_si_001.pdf]

Supporting Information

Molecular Design of a Metal–Nitrosyl Ferroelectric with Reversible Photoisomerization

Wei-Jian Xu,*† Mao-Fan Li,‡ Ana R. Garcia,‖ Konstantin Romanyuk,§ José M. G. Martinho,‖ Pavel Zelenovskii,† Alexander Tselev,§ Luís Verissimo,† Wei-Xiong Zhang,*‡ Xiao-Ming Chen,‡ Andrei Kholkin,*§ and João Rocha*†.

† Department of Chemistry & CICECO-Aveiro Institute of Materials, University of Aveiro, 3810-193 Aveiro, Portugal

‡ MOE Key Laboratory of Bioinorganic and Synthetic Chemistry, School of Chemistry, Sun Yat-Sen University, Guangzhou 510275, China.

§ Department of Physics & CICECO-Aveiro Institute of Materials, University of Aveiro, 3810-193 Aveiro, Portugal

‖ CQE- Centro de Química Estrutural, Institute of Molecular Sciences and Department of Chemical Engineering, Instituto Superior Técnico, University of Lisbon, 1049-001 Lisbon, Portugal.

Experimental

Synthesis

All chemicals were commercially available and used without further purification. Synthesis of $Ag_2[Fe(CN)_5NO]$ was reported by E. Reguera et al.[1] Compound **1** was prepared by a reaction of $Ag_2[Fe(CN)_5NO]$ (10 mmol) and dimethylamine hydrochloride (10 mmol) and piperidine hydrochloride (10 mmol) in 8 mL of deionized water. After the AgCl precipitate was filtrated, reddish-brown flaky crystals were obtained after the solution evaporated for several days. Yield: 87% based on $Ag_2[Fe(CN)_5(NO)]$. Elemental analysis, calcd (%) for **1** ($C_{12}H_{20}FeN_8O$): C, 41.39; N, 32.18; H, 5.79. Found, C, 40.99; N, 31.77; H, 5.42.

X-ray Crystallographic Analysis

The *in-situ* variable-temperature single-crystal diffraction intensities data were collected on a Bruker Smart APEX diffractometer equipped with Mo Kα sealed tube (λ = 0.71073 Å). The APEX3 software package was used for data collection, cell refinement, and data reduction. Using Olex^2 program,[2] the structures were solved by using Intrinsic Phasing with the SHELXT structure solution program and using full-matrix least-squares method with the SHELXL refinement program.[3] Non-hydrogen atoms were refined anisotropically and the positions of the hydrogen atoms were generated geometrically. The crystal data and structure refinement results for **1** are listed in Table S1. Powder X-ray diffraction (PXRD) patterns (Cu-$K_{\square}$, λ = 1.54184 Å) were collected on Panalytical Empyrean with Cu-Kα X-ray radiation (40 kV, 45 mA).

Elemental analysis

Elemental analyses for C, N, and H were performed with a Truspec Micro CHNS 630-200-200 elemental analyzer.

Thermal Analysis

Differential Thermal Analysis (DTA) and thermogravimetric analysis (TGA) were carried out on a Hitachi NEXTA STA300 with a heating rate of 10 K min^{-1} from 298 to 673 K under a nitrogen atmosphere. Differential scanning calorimetry (DSC) was carried out on a TA DSC Q2000 instrument under a nitrogen atmosphere in aluminum crucibles with heating and cooling rates of 10 K min^{-1} from 195 to 405 K.

Dielectric and *P*−*E* hysteresis loop measurements.

The dielectric measurements were carried out on a Keysight E4990A impedance analyzer at 16 frequencies from 500 Hz to 2 MHz, with an applied voltage of 1.0 V and a temperature sweeping rate of 3 K min^{-1} approximately in the range of 80–450 K in a Mercury iTC cryogenic environment controller of Oxford Instrument. The powder sample of **1** was ground and pressed into tablets under a pressure of around 5 GPa. The pressed-powder pellets were deposited with a magnetic sheet used as an electrode. *P-E* hysteresis loops measurements of **1** were done on ~2.0 x 1.5 x 1.5 mm^3 sized single crystal sample with silver paste electrodes by using a TF analyzer (TFA-1000).

SHG Measurement.

Variable-temperature SHG experiment was executed by Kurtz-Perry powder SHG test using an Nd:YAG laser (1064 nm) with an input pulse of 570 V under a programmable cryogenic cooling system.

Infrared (IR) spectroscopy.

KBr pellets of of compound **1** were mounted in a liquid nitrogen-cooled cryostat to allow laser irradiation without changing the optical geometry. The samples were irradiated with 405 nm light from a 25-mW continuous diode laser (BH, New York during 60 min. The spectra of the samples were obtained at increasing temperatures, from 77 K to 280 K, on a Bruker V70 FTIR spectrometer with an MCT wide band detector. In the investigation of photoisomerization reversibility, the spectra were recorded after alternating irradiation of the sample with 405 nm for 50 minutes followed by irradiation at 800 nm for 5 min (800-mW power Ti: sapphire laser). The spectra were recorded between 4000 cm^{-1} and 1200 cm^{-1}, with 4 cm^{-1} resolution (cut-off of the cryostat windows at 1000 cm^{-1}).

PFM measurements.

Imaging of the topography, domain configurations with high spatial resolution and ferroelectric switching were carried out by scanning probe microscopy (SPM) technique using piezoresponse force microscopy (PFM) mode implemented in the NTEGRA Aura scanning probe microscope (NT-MDT, Russia) equipped with the HF2LI lock-in amplifier (Zurich Instruments, Switzerland), Krohn-Hite wideband amplifier (7602M, U.S.A) and probes for PFM measurements (High-resolution silicon cantilevers of Tap 190E-G series (Budget Sensors) with Cr/Pt conductive coating with a resonance frequency of 190 kHz and a force constant of 48 N/m). PFM measurements were done with an ac voltage frequency of 21 kHz and an amplitude of 2V (PFM loops) and 4V, 8 V (PFM imaging).

Theoretical calculation.

Quantum chemical calculations were performed in the HyperChem 7.01 package using a semi-empirical PM3 method including the restricted Hartree–Fock approximation. Convergence limit was set 10^{-8}, iteration limit 32767. The calculations were performed for unit cell of **1** (Figure S1a,b) and individual anions and cations (Figure S1c-e). The atomic positions corresponding to the unit cell of **1** in the ground state were experimentally determined at 100 K by single crystals X-ray diffraction. The unit cell was first generated using CCDC Mercury software[4] and then transferred to HyperChem for calculations (Figure S1a,b). The metastable states were obtained by manual modification of nitroprusside anions in the same unit cell. The bond lengths and angles of nitroprusside anion in metastable phases were taken from reference.[5] Only the configuration of nitroprusside anions was changed; the positions of other molecules were constrained. No additional geometry optimization of the modified unit cell was done.

The calculations provided the dipole moments of the unit cell. The unit cell polarization, P [C/m^2], was determined using equation: $P = 3.33556255 \times D/V$, where D is the total dipole moment in Debye, and V is the unit cell volume in $Å^3$ determined from the X-ray analysis (Table S1). The numerical coefficient represents the combination of conversion factors to SI units. The obtained dipole moments and polarizations are presented in Table S1.

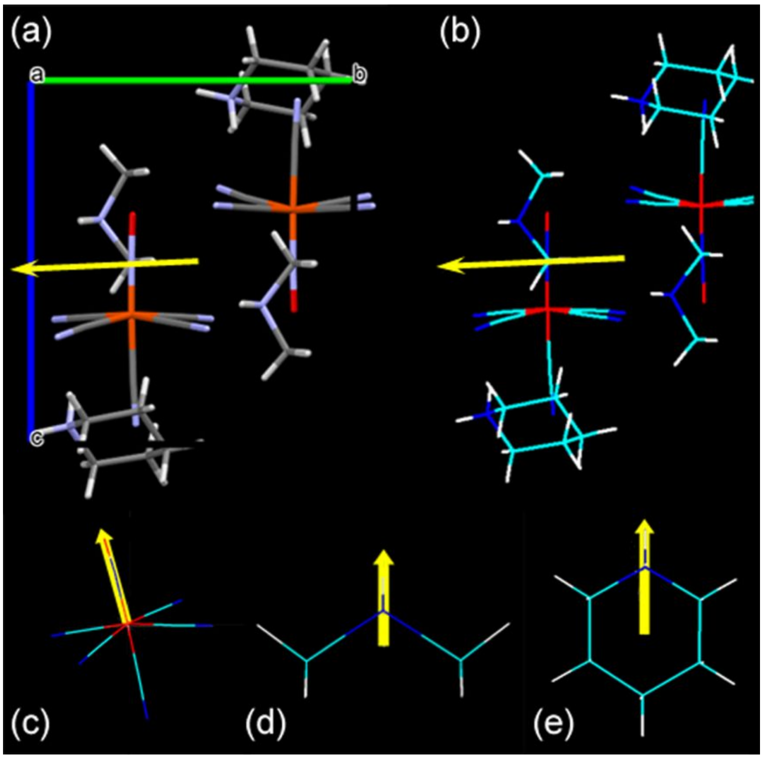

Figure S1. Atomic configurations used for quantum chemical calculations. (a) The unit cell of **1** constructed in CCDC Mercury, (b) its representation in the HyperChem workspace, and (c-e) individual molecules used in calculations: (c) nitroprusside anion, (d) DMA and (e) PIP cations. Yellow arrows show the orientation of the calculated dipole moments.

Table S1. The calculated values of the dipole moments of cations and anions in **1** and the total polarization of the unit cell.

Temperature (K)	100	298
Dipole moments (D)		
PIP	0.79	0.70
DMA	0.66	0.66
$[Fe(CN)_5(NO)]^{2-}$	10.72	10.71
Cationic sublattice	1.86	0.78
Anionic sublattice	0.54	0.68
Unit cell polarization (μC/cm^2)		
Total	7.66	6.47
Along *b* axis	7.64	7.26

Table S2. Crystal data and structure refinement parameters for **1** in the LTP, ITP, and HTP.

Compound	$(Me_2NH_2)(C_5NH_{12})[Fe(CN)_5(NO)]$ (**1**)			
Phase	LTP		ITP	HTP
Crystal system	monoclinic		monoclinic	orthorhombic
Space group	$P2_1$		$P2_1/m$	*Bmmb*
Temperature (K)	100(2)	298(2)	329(2)	385(2)
a (Å)	9.0562(8)	9.0168(12)	8.8300(13)	8.802(3)
b (Å)	9.0881(9)	9.2002(10)	9.2390(12)	9.312(3)
c (Å)	10.7238(9)	10.9971(16)	11.3402(18)	21.820(9)
β (°)	107.767(3)	107.636(5)	108.870(5)	90
V (Å^3)	840.51(13)	869.4(2)	875.4(2)	1788.4(11)
Z	2	2	2	4
D_c (g/cm^3)	1.376	1.330	1.321	1.293
μ (mm^{-1})	0.911	0.881	0.875	0.857
F_{000}	364.0	364.0	364.0	728.0
$R_1{}^a$ [$I > 2\sigma(I)$]	0.0386	0.0372	0.0630	0.0548
$wR_2{}^b$ [$I > 2\sigma(I)$]	0.1198	0.0844	0.1685	0.1527
$R_1{}^a$ (all data)	0.0397	0.0504	0.1021	0.0769
$wR_2{}^b$ (all data)	0.1208	0.0919	0.2093	0.1884
GOF	1.174	1.033	1.051	1.116
$\Delta\rho^c$, e/Å^3	1.00/-0.75	0.45/−0.40	0.81/-0.40	0.40/−0.37
Flack	0.014(5)	0.003(10)	/	/
CCDC	2240287	2240288	2240286	2240289

$^aR_1 = \sum||F_o|-|F_c||/\sum|F_o|$; $^bwR_2 = [\sum w(F_o^2 - F_c^2)^2/\sum w(F_o^2)^2]^{1/2}$. $^c\Delta\rho$, Maximum and minimum residual electron density.

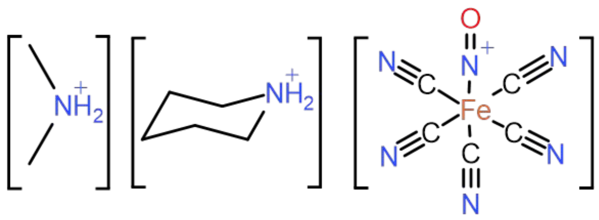

Scheme S1. The structural formula of **1**.

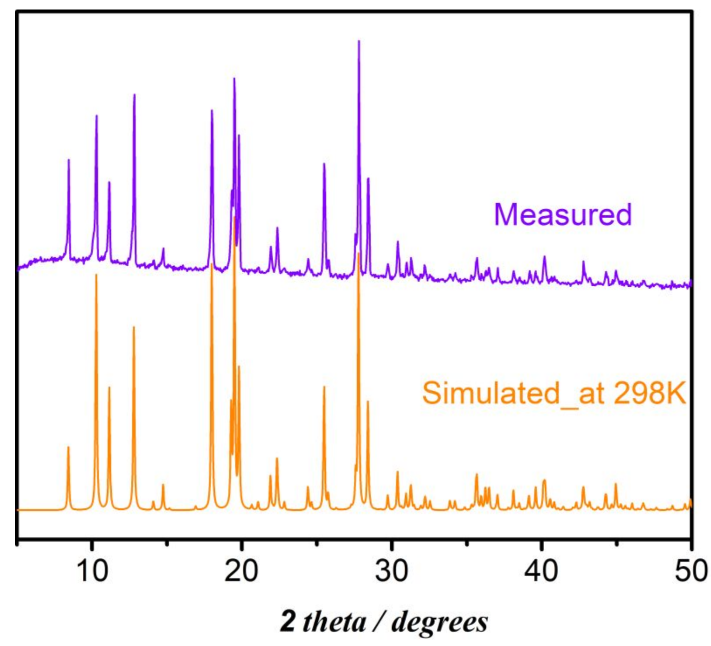

Figure S2. The powder XRD patterns confirmed the phase purity of the as-synthesized sample **1**.

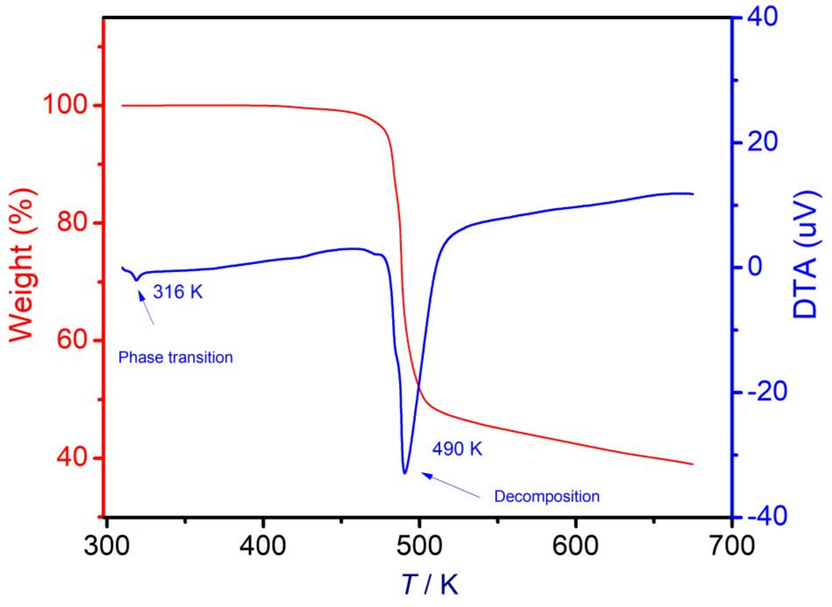

Figure S3. TGA and DTA thermograms of **1**. It should be note that DTA is a less sophisticated technique than DSC, the small entropy change of phase transition at 369 K is not detectable.

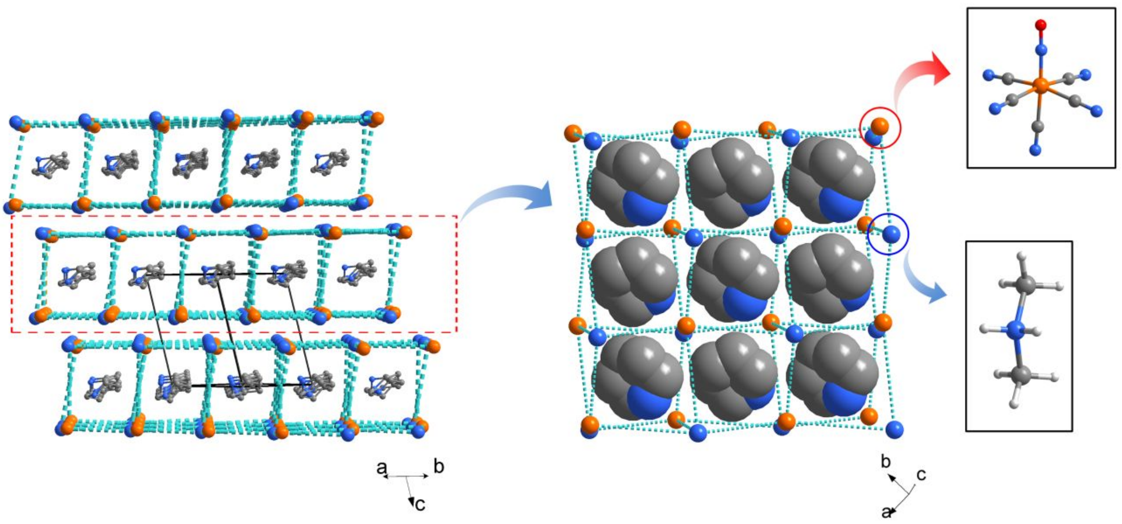

Figure S4. The simplyfied structure of **1** at 100 K (LTP). $[Fe(CN)_5NO]^{2-}$ and DMA cation are simplified as orange and blue spheres, respectively.

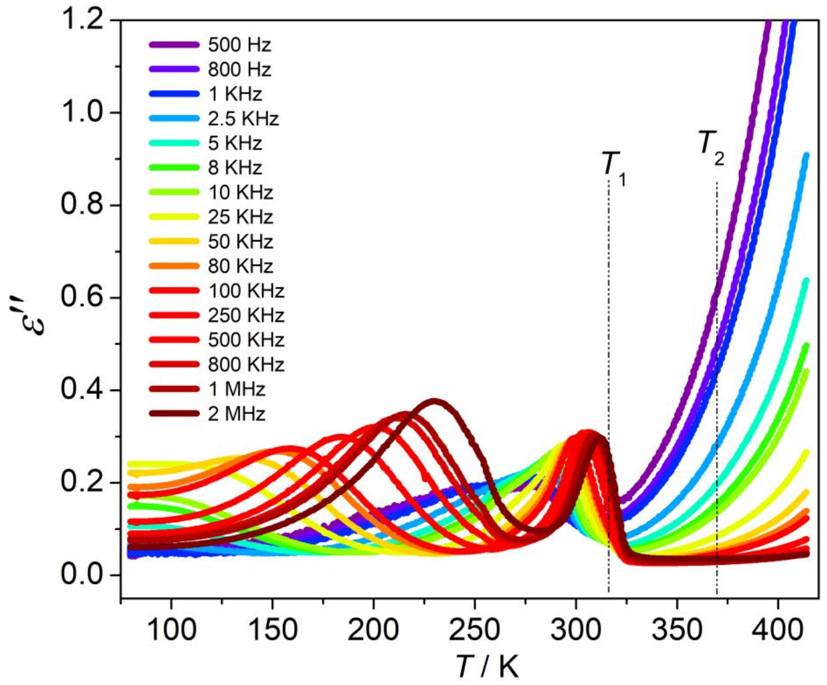

Figure S5. Imaginary part (*ε''*) of the complex dielectric constant measured on the polycrystalline pellet of **1** at various frequencies in the heating process.

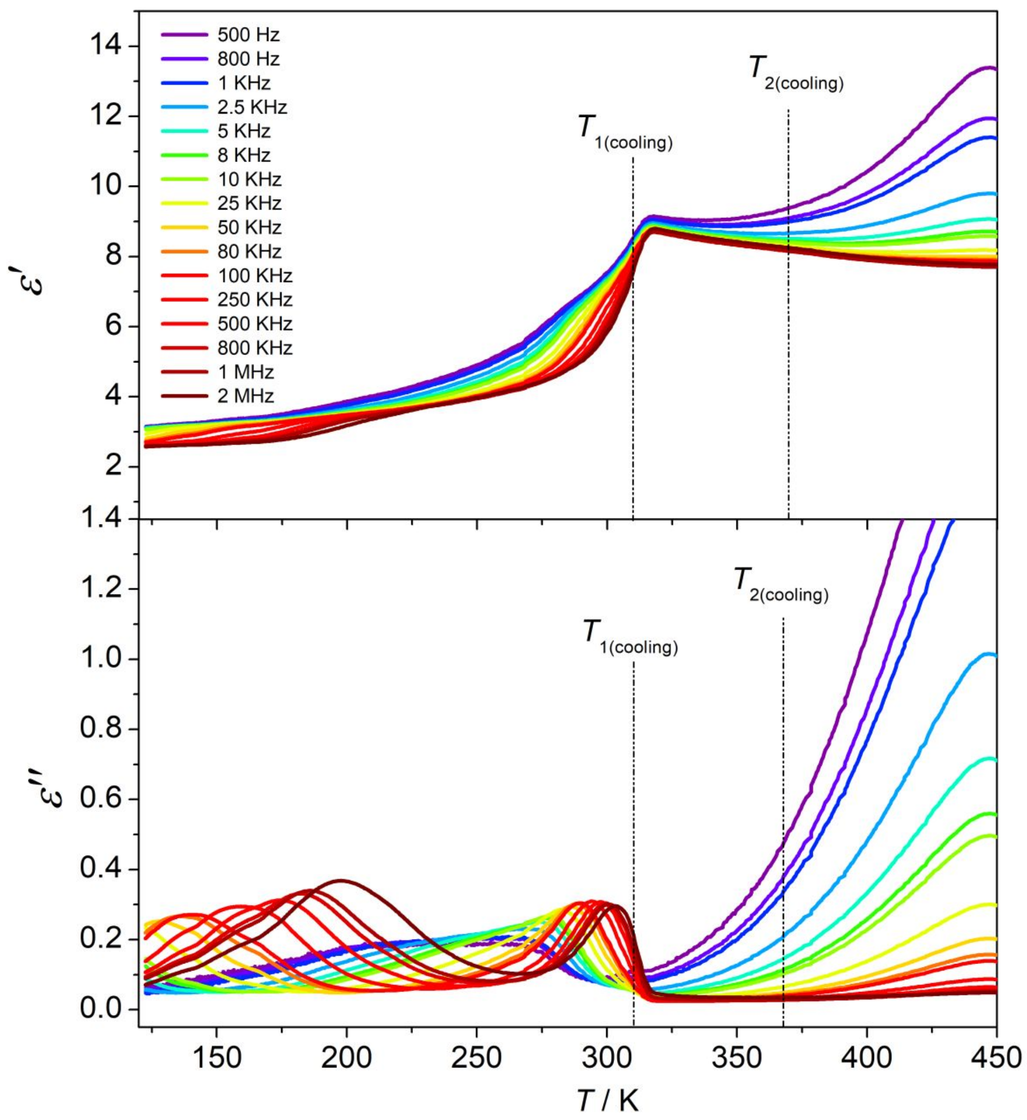

Figure S6. Temperature-dependent (a) real part (*ε'*) and (b) imaginary part (*ε''*) of the complex dielectric constant measured on the polycrystalline pellet of **1** at various frequencies in the cooling process.

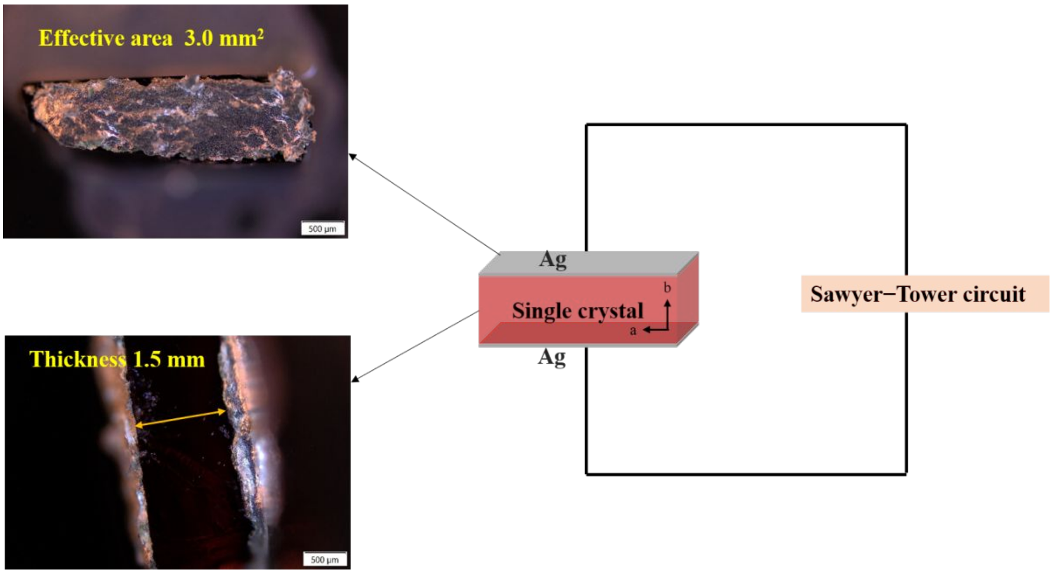

Figure S7. The single crystal sample used in the *P*-*E* loop measurement.

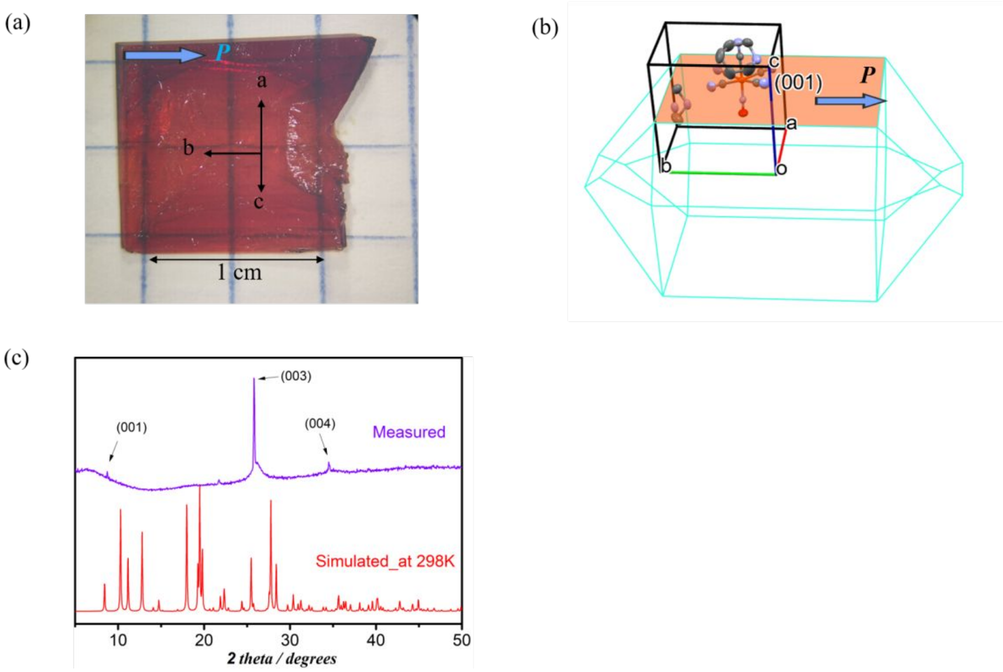

Figure S8. (a) Crystal morphology of **1**. (b) The growth habit of **1** was simulated by Bravais−Friedel−Donnay−Harker (BFDH) method. (c) X-ray diffraction (XRD) pattern of the single crystal of **1**.

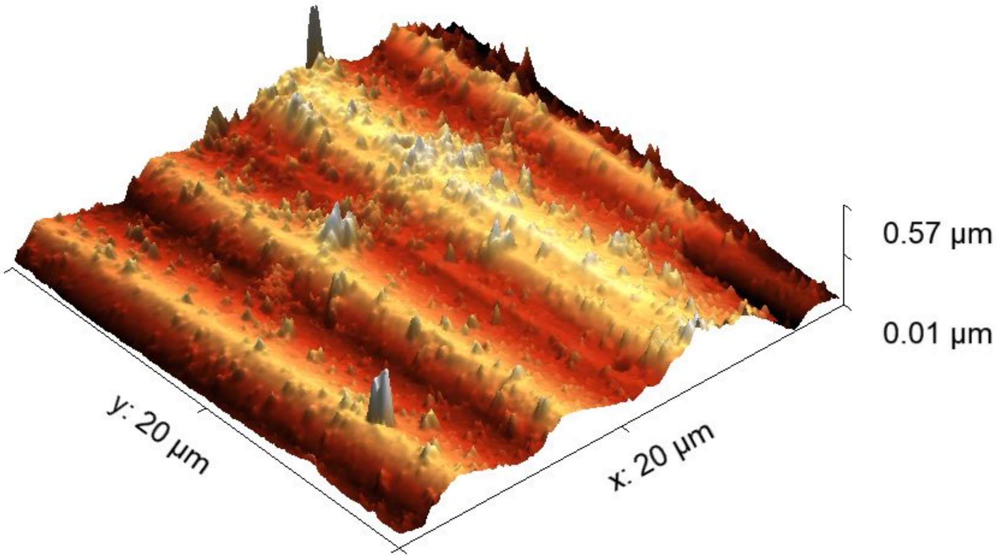

Figure S9. 3D representation of an AFM topography image of a sample of **1** annealed at 373 K for 5 minutes, that is, above the temperature of the transition to HTP. The image reveals a system of alternating ridges and furrows evidencing sample twinning upon transition from orthorhombic to monoclinic crystal structures. Corrugation of the surface appears due to opposite signs of the spontaneous strain (monoclinic distortions of the higher-symmetry orthorhombic structure) in adjacent ferroelastic domains on cooling from the annealing temperature. The monoclinic distortion at the HTP-to-ITP transition is significantly larger than at the ITP-to-LTP transition. As a result, the clapping angle, that is, the angle needed to turn one of the adjacent ferroelastic domains towards the other to preserve the material integrity is much larger at the HTP-to-ITP transition than at the ITP-to-LTP transition.[6] As can be determined from the profile of the surface corrugation in the image, the clapping angle between adjacent ferroelastic domains is between about 10° and 14°, while the values calculated with the use of the data of Table S1 yield 12.7° for the complete HTP-to-LTP transition. In turn, for the ITP-to-LTP transition, the clapping angle is 3.8°. This value would result in a topography with a corrugation amplitude comparable or below the surface feature height seen in the topography images in Figure 5 of the main text, which makes it difficult to reveal twinning in the topography PFM images displayed in the main text.

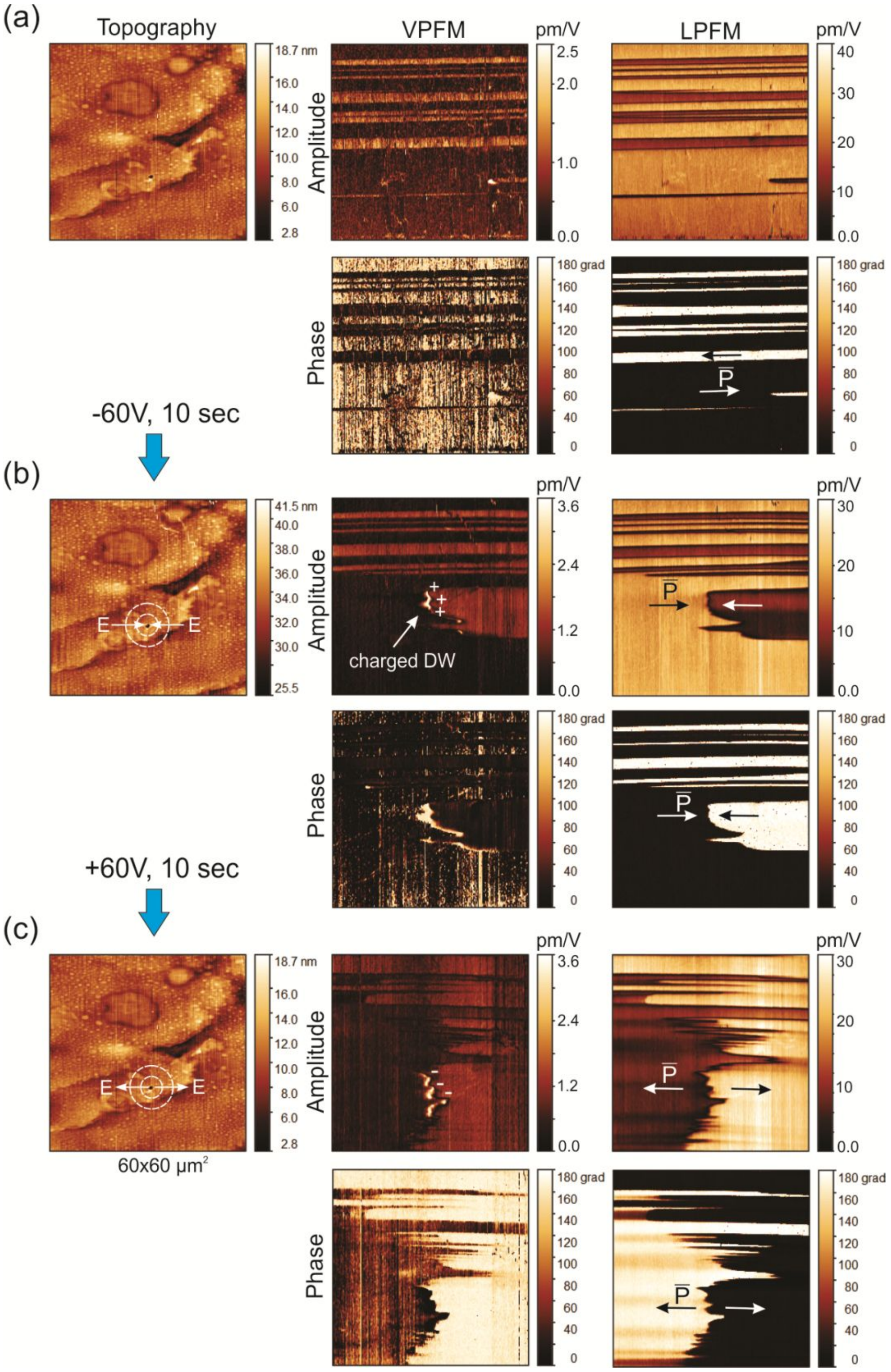

Figure S10. Domain switching by application of a voltage ±60 V for 10 s at the position marked by the dashed circles in the topography images. (a) Pristine topography, vertical PFM, and lateral PFM images before voltage application. (b) Topography, vertical PFM and lateral PFM images after application of a voltage of –60 V. (c) Topography, vertical PFM and lateral PFM images after application of +60 V. Image size 60x60 μm^2.

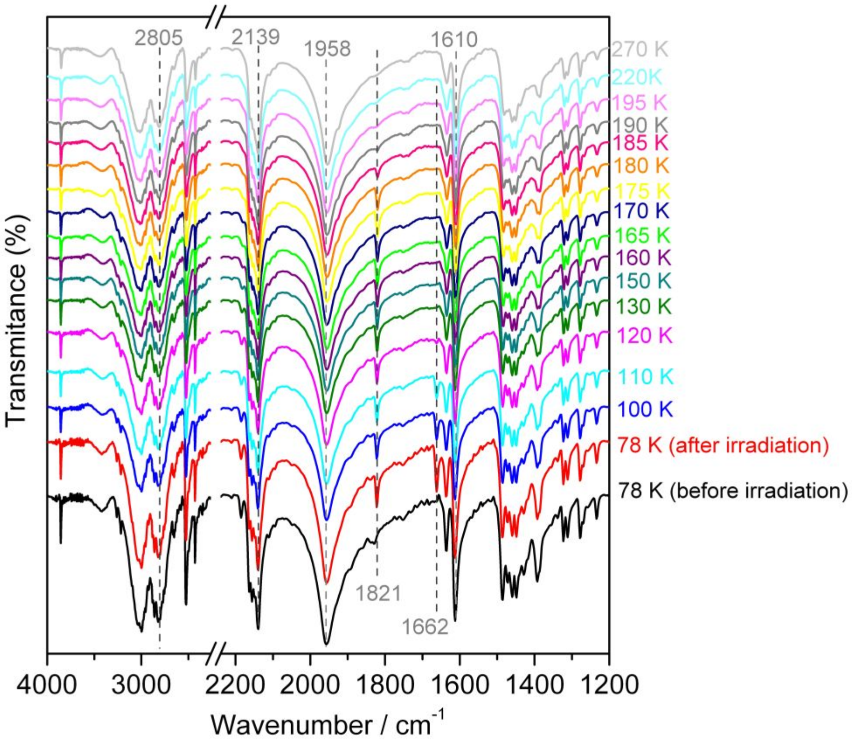

Figure S11. FTIR spectrum of **1** before and after 405 nm light irradiation at the specified temperatures.

References

[1] J. Rodríguez-Hernández, L. Reguera, A. A. Lemus-Santana, E. Reguera, *Inorg. Chim. Acta* **2015**, *428*, 51-56.

[2] O. V. Dolomanov, L. J. Bourhis, R. J. Gildea, J. A. K. Howard, H. Puschmann, *J. Appl. Crystallogr.* **2009**, *42*, 339-341.

[3] G. Sheldrick, *Acta Cryst.* **2015**, *C71*, 3-8.

[4] C. F. Macrae, P. R. Edgington, P. McCabe, E. Pidcock, G. P. Shields, R. Taylor, M. Towler, J. van de Streek, *J. Appl. Crystallogr.* **2006**, *39*, 453-457.

[5] M. D. Carducci, M. R. Pressprich, P. Coppens, *J. Am. Chem. Soc.* **1997**, *119*, 2669-2678.

[6] P. Mokrý, J. Fousek, *J. Appl. Phys.* **2005**, *97*, 114104.
